# Career choices and what influences Nepali medical students and young doctors: a cross-sectional study

**DOI:** 10.1186/1478-4491-11-5

**Published:** 2013-02-08

**Authors:** Bruce W Hayes, Rabina Shakya

**Affiliations:** 1 Nick Simons Institute, PO Box 8975, Kathmandu EPC 1813, Nepal; 2 Patan Academy of Health Sciences and Patan Hospital, PO Box 252, Kathmandu, Nepal

**Keywords:** Career choice, Medical students, Generalists, General practitioners

## Abstract

**Background:**

Nepal, as a nation with limited resources and a large number of poor people, needs far more well-trained, committed general practitioners. The aim of this study was to understand medical career choices and the factors that influence medical students’ and young doctors’ career choices in Nepal and to understand what would encourage them to work in rural areas as generalists.

**Methods:**

This was a cross-sectional study of 1137 medical students (first and final year) and young doctors (interns and residents) from six medical colleges in Nepal who completed a voluntary questionnaire, with some also participating in structured focus groups – 170 first years, 77 final years and 80 graduates – with an additional 28, 44 and 49 written responses respectively.

**Results:**

Without selective admissions policies, 41.7% (464/1112) of respondents had a rural background – most significant in Year 1 students, males and in colleges outside of Kathmandu. Of the respondents, 569 (50.9%) had a specialty choice starting medical school – the greatest proportion in Year 1. Medicine (especially cardiology) and surgery (particularly among males) were most significant choices at all stages. Only five participants initially and four during their course chose general practice. There appears no interest in, recognition of, significant exposure to, or role models in general practice.

Serving the sick, personal interest and social prestige were the most significant influencing factors – consistent across all groups. Course availability was also a factor. To attract doctors to work in rural areas most respondents affirmed the need for a good salary, infrastructure and facilities, scholarships and career development opportunities.

**Conclusions:**

Challenges include raising generalists’ profiles within the medical community, government and patient community; changing undergraduate curricula to include greater exposure to good models of rural generalist practice; and providing incentives and attractions for post-graduate training and service.

## Background

Nepal faces a shortage of appropriately trained health care workers including doctors to work outside Kathmandu and the other main centres, despite increasing numbers of medical graduates. In 2009, of the 8,118 doctors working in Nepal, Kathmandu’s doctor density was estimated to be 25 times more than in rural Nepal [1].

This is not limited to Nepal; The World Health Organization (WHO) states “one of their most complex challenges is ensuring people living in rural and remote locations have access to trained health workers” [2].

WHO recommends targeted admission policies to enroll students with a rural background; locating health professional schools, campuses and family medicine residency programmes outside of capitals and other major cities; and exposing undergraduate students of various health disciplines to rural community experiences to increase the likelihood of graduates choosing to practise in rural areas. Revision of undergraduate and postgraduate curricula to include rural health topics to enhance the competencies of health professionals working in rural areas, and thereby increasing their job satisfaction and retention, is also recommended [2,3]. Thistlewaite et al. [4] and Henry et al. [5] confirmed in their literature reviews that prior rural residence is the strongest predictor of choice of a rural career but extended rural exposure during medical training and role models also have a significant impact on choice of rural career.

Countries with a strong primary health care system have lower premature mortality and better health care outcomes than those with a specialist focus [6], and care by a general practitioner reduces disparities in health (the gap between rich and poor), reduces the effect of income inequality on health and improves self-rated health [7]. With this evidence in mind the World Health Assembly in May 2009 adopted a resolution urging member states to “accelerate action towards universal access to primary health care” and “to train and retain adequate numbers of health workers . . . including . . . *family physicians*. . .” [8].

The reasons medical students choose their careers are complex. Factors shown to be associated with choosing family medicine include medical school characteristics (proportion of faculty who are family physicians), personal interactions and lifestyle preferences, personal fit and workforce factors, including expected income, prestige, job opportunities, longitudinal care and societal need [4,9,10]. Career preference at the time of entering medical school may be a significant predictor of students' eventual career choices, and as a result, defining the factors that influence career choice at the start of medical school is important [9]. Mahoney et al. [11] suggest that there is a critical period during the end of medical school training and the first two years after qualification in which career intentions change and that career advice should be available during this time.

In Nepal, the General Practice Postgraduate Programme (MDGP) which started in 1982 was the first postgraduate programme in Nepal. It is a 3-year programme, initially started in the Institute of Medicine (IOM) and also conducted in BP Koirala Institute of Health Sciences (BPKIHS) since 2001 and the National Academy of Health Sciences (NAMS) since 2005. In previous studies among graduated MDGPs in Nepal [12], having a spouse who grew up outside Kathmandu and whether the doctor had ever been a Health Assistant were the statistically significant factors in whether a general practitioner was currently working outside Kathmandu.

In a later study among Institute of Medicine (IOM) graduates with bachelor of medicine and bachelor of surgery(MBBS) [13], lower class rank, paramedical background, and rural upbringing were all factors significantly associated with a doctor’s remaining in Nepal, and moreover, with working outside the capital city. This study of 710 (97.7%) of the 727 graduates from 1983 to 2004 also showed that 256 (36.1%) were working in foreign countries. Of 256 working abroad, 188 (73%) were in the United States. Students from later graduating classes were more likely to be working in foreign countries. In a more recent study of career intentions among final year medical students from four Nepal medical schools, students who indicated a greater likelihood of practicing in rural areas were more likely to be male, to have gone to a government secondary school, to have been born in a village, or to have received a scholarship from the Ministry of Education that requires rural service [14].

Nepal, as a nation with limited resources and a large number of poor people needs far more well-trained, committed general practitioners. In 2012, there were still fewer than 200 Nepali MDGPs for a country with a population of 26,620,809 according to the 2011 census [15]. With this need, the aim of this study is to understand what career preferences medical students and young doctors have at the beginning of, during and after medical school; to explore factors that influence medical students’ and young doctors’ medical career choices in Nepal; and to look at what would encourage them, including types of generalist training, to work in rural areas as generalists.

## Methods

This was a cross-sectional study of medical students (first year and final year) and young doctors (interns and residents) from six medical colleges in Nepal. Three colleges were in Kathmandu and three were outside Kathmandu. One college that was approached declined our request. The colleges were contacted and an outline of the study and the questionnaires were submitted to obtain approval from the college leadership and administration. We only went in to the colleges with the approval of the leadership of the college.

After obtaining consent from the college leadership, we went into classes and gave a paper-based personal questionnaire to all year 1 and final year students. Through a doctor contact working in the college hospital, we gave the questionnaires to interns and residents working there with the aim to get all those available to complete the questionnaire. This was done voluntarily. This sought demographic data and rating of factors influencing their career choice of medicine and then in their postgraduate choices using a Likert rating scale of 1–5.

For further clarification about career influences and specific issues of rural generalist practice, we organized focus groups from these classes and groups. These focus groups varied in size from 7 to 60 participants of interested students (first year and final year) and doctors and were conducted with a structured questionnaire. The aim was to hear directly from participants about factors influencing their career choice and about choosing rural practice. Participation was entirely voluntary, and we offered a lunch box to those participating. In one urban college the entire first-year class of 60 stayed for the discussion. Because of the difficulty in getting interns and residents together we also organized a dinner for the Kathmandu residents from the three colleges for the purpose of organizing focus groups. In some colleges where people could not stay for groups they voluntarily gave written responses to the structured questions, which were reviewed and added to the focus group comments.

We recorded all focus group discussions on tape recorders and individually analysed the recordings to summarize the main responses. Each of us then independently reviewed all these data, summarized the responses under each question and grouped respondents according to their year and whether they had attended or were attending a rural or urban college. In total we had 170 first years respondents (9 groups), 77 final year respondents (6 groups) and 80 graduates (8 groups) with additional 28, 44 and 49 written responses, respectively. This was from a possible 710 first year students, 510 final year students and 600 graduates.

There was a mix of quantitative and qualitative data. We used Statistical Product and Service Solution (SPSS) version 12 (2004) for data analysis. Demographic data were presented as percentages and means. Comparisons used the Chi squared test or Fisher’s exact test (where there were fewer than five in a cell in the 2 × 2 table), or Student *t* test for continuous data. Significance was set at <0.05.

## Results

### Demographics

**Figure 1:**
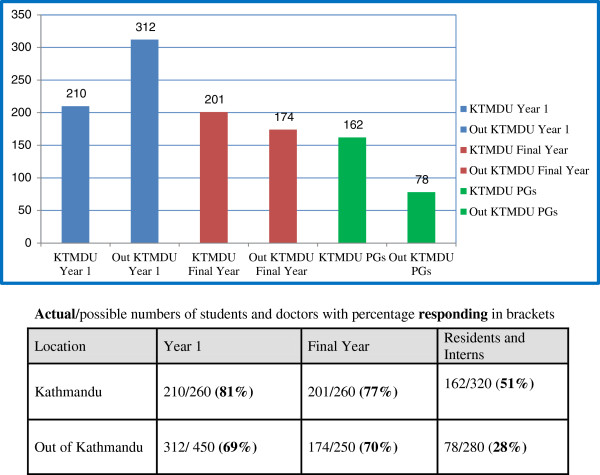
**Respondent numbers by year of study and place of medical college. **Actual/possible numbers of students and doctors with percentage responding in brackets.

**Figure 2:**
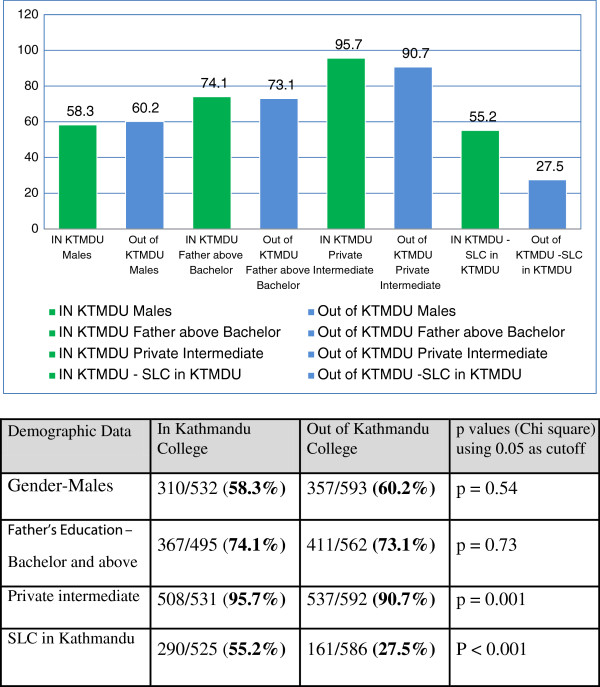
Demographic comparison of colleges in Kathmandu and out of Kathmandu by percent.

There were a total of 1137 of a potential 1820 responses or 62.5% (some incomplete information in varying amounts as seen in the different figures) from different colleges and years as shown in Figure 1. There was no significant difference in gender distribution between in- and out-of-Kathmandu colleges (Figure 2). The age distribution of students and doctors at each stage was similar across the institutions. Only five doctors were married. There were no married students.

Overall 41.7% (464/1112) of respondents had a rural background using the place where School Leaving Certificate (SLC) was undertaken as marker of background (Table 1).

**Table 1:** Background using place where School Leaving Certificate (SLC) done

**Demographic factors**	**In Kathmandu**	**Out of Kathmandu**	**Other (mainly India)**	***P *values (Chi square tests)**
**Place of SLC**	451/1112 **(40.6%)**	464/1112 **(41.7%)**	197/1112 **(17.7%)**	
**Male (59.5%)**	214	337	105	<0.001
**Female (40.5%)**	234	120	92	
**Year 1 (46.3%)**	198	251	66	<0.001
**Final year (32.9%)**	152	120	94	
**Postgraduate (20.8%)**	101	93	37	
**College in Kathmandu (47.3%)**	290	198	37	<0.001
**College out of Kathmandu (52.7%)**	161	265	160	
**Father’s education –intermediate and below (26.6%)**	91	162	24	<0.001
**Father’s education –bachelor and above (73.4%)**	331	274	160	
**Intermediate private (93.6%)**	444	435	153	<0.001
**Intermediate government (6.4%)**	5	25	40	

Further demographic data are presented in Figure 2 (with comparisons of location of colleges) and Table 1 (with comparisons of background according to place where SLC was undertaken).

### Choices

**Figure 3:**
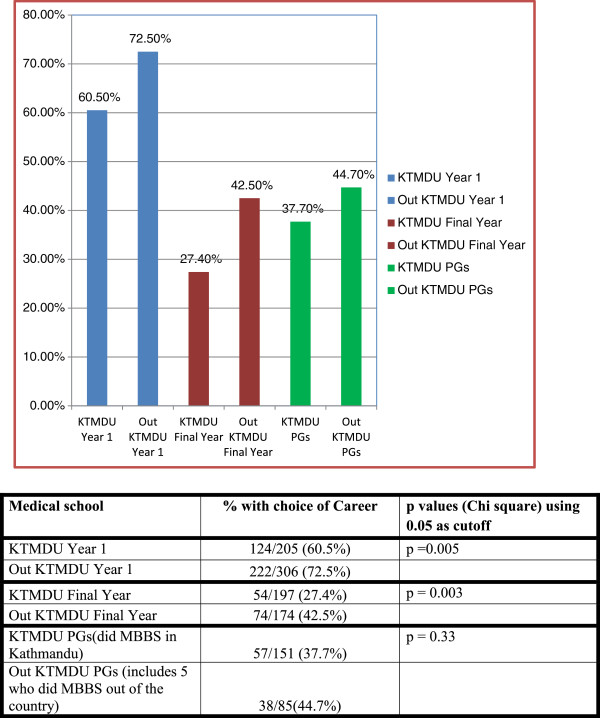
Groups with choice of career at start of medical school studies according to place of undergraduate medical school.

Overall, 50.9% (569/1118) had made a choice of specialty at the start of medical school – the greatest proportion was those in year 1, those students from colleges outside of Kathmandu (Figure 3), and those with a rural background (Table 2).

**Table 2:** Choice at start by background according to place of School Leaving Certificate (SLC)

**Place of SLC**	**In Kathmandu**	**Out of Kathmandu**	**Other (mainly India)**	***P *value**
**Have choice**	201/446 (45.1%)	254/454 (55.9%)	104/194 (53.6%)	*P* = 0.004

**Figure 4:**
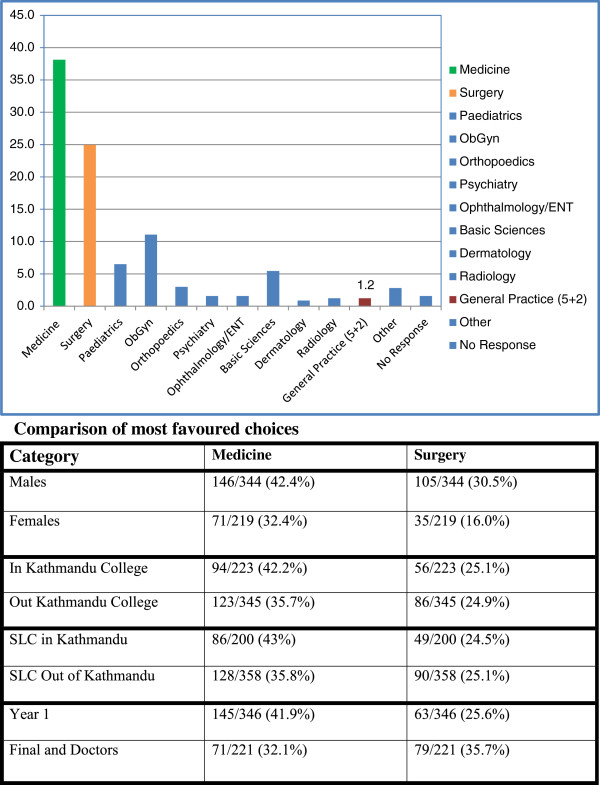
**Specific career choices by specialty. **Comparison of most favoured choices.

Among those who had a choice of specialty, a choice of general practice was very low (as shown in Figure 4). Only five Nepalis chose general practice – four from rural backgrounds, with one in first year, two in final year, and two among the interns and residents. Two were from a college outside Kathmandu and three were from a college in Kathmandu. There were another two international students who chose general practice.

The favoured choices were as indicated below:

• Medicine (217 or 38.1%) - Cardiology (65) and Neurology (34) were significantly mentioned.

• Surgery (142 or 25.0%).

• Obstetrics and Gynaecology (63 or 11.1%); 58 were female and this specialty represented 26.5% of females’ choices.

Small, but increasing numbers of those with a choice at the beginning of medical school reported a change in their choice during the course of their medical training: Year 1, 24/337; Final Year, 26/119; and Interns/Residents, 35/92; with medicine (24) and surgery (10) the top two choices. No one changed to general practice.

**Figure 5:**
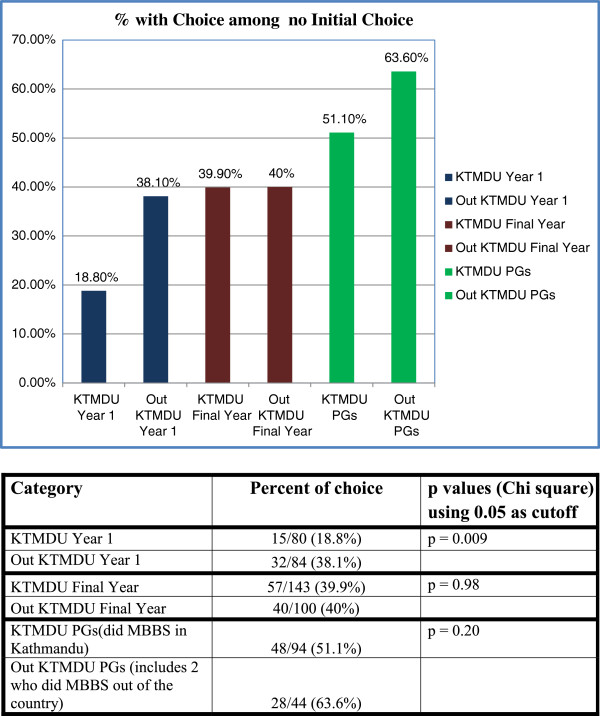
Group with choice of career now among those who had no choice at start.

**Figure 6:**
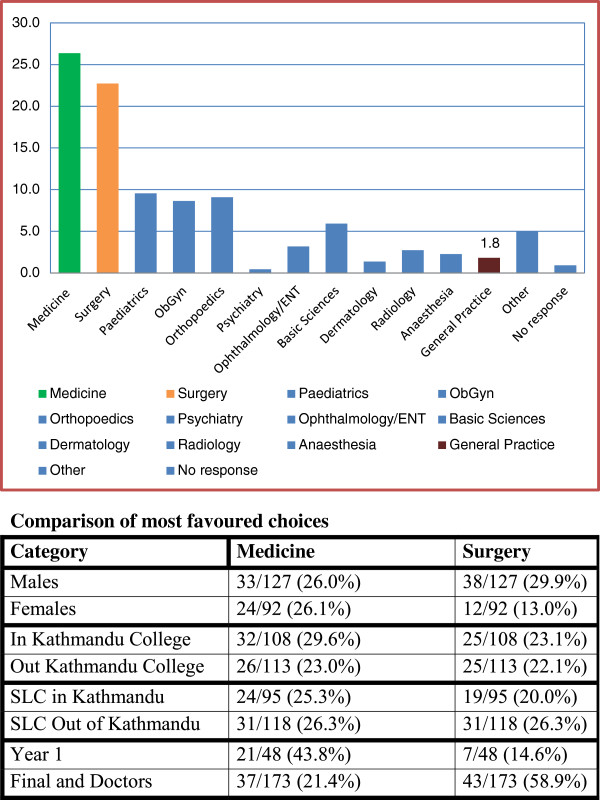
**Specific career choice by specialty among those who had no initial choice. **Comparison of most favoured choices.

Among the group who had no choice initially, 220 now had a choice with increasing proportions among the interns and residents (Figure 5). Among this group (of 220), very few (four) chose general practice – all were males from Kathmandu colleges with two in first year (both with Kathmandu background), none in final year, and two among the interns/residents (one from Kathmandu and one from Indian background) (Figure 6).

The main interests were similar to those with choice from the start though with a wider spread of choices (Figure 6).

• Medicine (58 or 26.4%) - Cardiology (14) and Neurology (5) were again mentioned.

• General Surgery (50 or 22.7%).

• Obstetrics and Gynaecology (19), Orthopaedics (20) and Paediatrics (21) were similar.

In all, 117/511 (22.9%) of first years, 146/371 (39.4%) of final years and 62/236 (26.3%) of the interns/residents remained undecided.

### Focus groups

In the focus groups, availability (what one could get into) was the strongest factor stated for choosing postgraduate courses - “being a woman, one cannot wait for years to get the specialty of their choice.” Not many in the groups had definitely chosen their postgraduate course as yet, reflecting more reliance on availability. There had not been exposure to talks or informational programs regarding specialties during their course of study, which they thought was necessary. Most (especially the more junior students) had not heard or understood about general practice. There was more community exposure among the doctors, but the consensus was that these “don't have good impact as there is lack of facilities, equipment, medicines, and awareness etc.” and generally exposure has had a negative effect. Also, most of the doctors had met MDGPs, but no one reported having seen a good role model.

They reported that though MDGP (postgraduate in general practice) seems useful and is especially needed in rural Nepal (“One gets to serve people who really need help” and can “practice all learnt”), MDGP is “not comparable to Masters in Medicine or Surgery (MD/MS) – it is like doing MBBS all over again". They reported that people want specialized doctors and MDGPs are “not a specialist” (“Jack of all trades and master of none”) and so there is poor recognition. Thus, having equal status as specialists was seen as very important, and the concept of seeing a general practitioner (GP) before a specialist was also seen as a way of increasing recognition. However, at present, they see “not much role because no one really thinks of going to a GP for consultation before they go for the specialist” and they reported that there is a perception that GPs are not respected by colleagues. Opportunities for GPs are only seen as good outside the Kathmandu valley (with the attendant inadequate salary, facility and infrastructure issues) with little role in the city apart from Emergency Departments. They expressed concern of being “stuck in rural areas”.

In general there was very little interest in diploma courses. Everyone was set on an MD (“a must to practice”) even in the face of the relatively low number of available places (estimated currently at about 20% of all Nepali MBBS graduates) and would keep trying for this. One group, the 2-year government scholarship holders showed some interest “if the diploma could be used as a stepping stone to advance their career” (e.g., if the diploma gave them an edge while competing for PG seats). A common theme among all was that for a diploma to have value it had to provide something for MD training or give some recognition (e.g., in government service). The bond holders were also the group most interested in distance programmes (generally not well known or understood) to help them continue study during their service period. If they couldn’t get into an MD programme, some were considering going abroad or doing a Masters in Public Health (MPH), but they were generally optimistic that there would be more places and that if they kept trying they would eventually get into an MD programme.

### Factors affecting choice

Table 3 gives the mean significance scores that respondents attributed to factors that might affect their choice of medicine and of postgraduate career. Means were compared between respondents of urban and rural background using the Student *t* test – only one comparison (opportunity for procedural work) reached statistical significance and is probably not practically significant.

**Table 3:** Mean rating of factors affecting choice (based on 5-point Likert Scale from 1-not significant to 5-very significant)

**FACTOR**	**Choosing medicine (MBBS**^**a**^**) (SLC**^**b **^**urban *vs *rural background)**	**Choosing PG**^**c **^**course (SLC urban *vs *rural background)**
Serve sick	4.4 (4.4, 4.4)	4.2 (4.19, 4.21)
Personal interest	4.22 (4.16, 4.27)	4.27 (4.29, 4.25)
Social prestige	4.15 (4.14, 4.18)	4.12 (4.15, 4.1)
Employment prospects	3.83 (3.79, 3.85)	3.88 (3.9, 3.87)
Best available course	3.62 (3.58, 3.67)	3.69 (3.7, 3.69)
Serve rural areas	3.47 (3.48, 3.47)	3.39 (3.39, 3.39)
Self/government employment	3.47 (3.42, 3.51)	3.58 (3.54, 3.62)
Scientific/research interest	3.46 (3.44, 3.49)	3.54 (3.56, 3.54)
Financial remuneration	3.25 (3.23, 3.27)	
Financial remuneration –during PG study	N/A	3.35 (3.41, 3.32)
Financial remuneration – after PG study	N/A	3.7 (3.67, 3.74)
Personal or family Illness	2.77 (2.81, 2.76)	2.74 (2.8, 2.7)
Books/films	2.74 (2.76, 2.74)	2.67 (2.62, 2.71)
Family pressure	2.60 (2.64, 2.58)	2.37 (2.37, 2.37)
Same doctor in family	2.56 (2.53, 2.58)	2.13 (2.13, 2.13)
Work satisfaction	N/A	4.35 (4.37, 4.33)
Opportunity for procedural work	N/A	3.72 (3.83, 3.65) *P* = 0.002
Work hours	N/A	3.55 (3.52, 3.58)
Pursuit of non-medical interests	N/A	2.69 (2.69, 2.7)

^a ^MBBS – Bachelor of Medicine, Bachelor of Surgery.

^b ^SLC - School Leaving Certificate.

^c ^PG - postgraduate.

In the focus groups, prestige, social service and personal interest were the main reasons students and young doctors stated that they had gone into medicine – consistent with the findings from the questionnaires. There were a few participants with parental influence or doctors in the family, but this was generally a small impact.

To attract doctors to work in rural areas most identified good salary (“much more than in Kathmandu”) and infrastructure/facilities (well-equipped and served facility and a good team because the “doctor can’t do it alone”) as key factors. Scholarship and career development opportunities (including Continuing Medical Education (CME) and higher education), special facility package for rural doctors, education for children, community awareness and cooperation about the available hospital services, government policy of transfer (“not being stuck in rural areas”), political stability and security, support by specialists/consultants and recognition of their services were also important for many.

## Discussion

In this sample of medical students and young doctors, there was already a significant number (41.7%) with an out-of-Kathmandu background (according to place of SLC) with no selective admissions policy in any of the colleges. This appears to provide less of an opportunity to increase rural practice than in western experience and as suggested by WHO [2,3]. Interestingly, the proportion of medical students and young doctors with an out-of-Kathmandu background was highest (48.7%) in the year 1 students and was significantly higher among males and in the colleges outside of Kathmandu (Table 1 and Figure 2).

Unsurprisingly, most of the respondents came from families with academic backgrounds (73.4% with father with bachelor level or above and with only 4.4% below SLC) though there was a significantly lower proportion in those from an out-of-Kathmandu background (62.8%) with no difference between Kathmandu and out-of-Kathmandu colleges (Figure 2). Also, most (93.6%) respondents had undertaken their intermediate study (equivalent of grade 12) through private colleges with a significantly higher proportion among those from Kathmandu and a lower proportion in those from India. The significantly higher proportion with a private intermediate education in those from Kathmandu colleges reflected the higher proportion of Indian students in out of Kathmandu colleges (Table 1 and Figure 2).

In assessing respondents with a specialty choice, we noted that current first years had the highest percentage of choice at the start of their medical school; there is a possibility that this could reflect clearer and more recent memory among them. Also, specialty choice was higher in rural college students in both first and final year (Figure 4). As expected with the passage of time, there was both more change in choice and increasing number who had made a choice since starting (Figure 5) among the interns/residents.

Not surprisingly, and probably consistent with the general community view of their prestige, medicine (especially cardiology) and surgery (particularly among males) were the most significant choices at all stages – at the start of course, when changing choices, and when making choices during the course. Medicine was favoured among males, among those from Kathmandu backgrounds and Kathmandu colleges, and among first years though less marked in those choosing during the course. Surgery was favoured among males, among final years, and among PGs without much difference in background and college. Consistent with social attitudes concerning care of females by females, as possible, Obstetrics and Gynaecology was most popular among females. In those participants who changed their choice and made a choice during the course there is a broader spread of subject choices, perhaps reflecting exposure during their course.

General practice was rarely chosen both at start (five Nepalis), among those who changed (zero), and those who had made a choice during their course (four). This is consistent with Huntington et al.’s study of final year students in Kathmandu [14] where 8/469 or 1.7% indicated a possibility of doing MDGP. Overall there has been very little exposure apart from some visits and camps to rural and community work in undergraduate courses. There is an urgent need in undergraduate courses for exposure to good GP role models and functioning rural facilities.

However a reasonable number of students and young doctors remain undecided, suggesting some scope for influence on their final career choice with what is clearly an enormous need to raise the general practice profile. Interestingly, the proportion of undecided respondents was lowest among the first year students (22.9%). In focus groups of the final-year students and doctors, clinical exposure and the role models of teachers in internship were suggested as being an important influence in choosing specialty. These groups reported that they saw role models and teachers as inspirational while their own interest and availability are greater influences on their specialty choice. This highlights the importance of exposure to good role models during the intern years as seen in the United Kingdom and Australia, which have a more similar system of PG courses some time after graduation [10,11].

Among the factors influencing career choices, serving the sick, personal interest and social prestige were stated to be the most significant. These factors were consistent across all groups whether urban or rural and at different career stages and whether making undergraduate and postgraduate choices. Personal interest became slightly more significant in postgraduate choice, and work satisfaction was the most significant for postgraduate choice. Family pressure, same doctor in family, books/films, and personal or family illness were the least significant, and again was consistent across all groups. This was confirmed in the focus groups.

Overall, lifestyle (work hours and pursuing other interests), which is significant in the western literature [4,16] was less significant in this Nepal group. Background showed no significant differences in factors affecting choice.

The influencing factors contrast with the Australian review where continuity of care, flexibility and hours, lifestyle, lots of variety and stimulating work, working with people, autonomy, prestige, skill mix, social status and holistic care were important attractions of general practice as a career [4]. There were some similarities to the unattractive factors for general practice found in the Australian review [4]; lack of support, not intellectually challenging, lack of time with patients, negative media coverage and lack of prestige were main factors. Clearly, both raising awareness and status of MDGPs as well as a career path is vital in attracting young doctors into general practice. One innovative suggestion to attract doctors into MDGP training was if the training was government sponsored after 1 year service of the required two bonded years for government sponsored MBBS graduates.

There is clearly still much to be done to attract doctors to rural areas as seen in the following comment: “For a fresh graduate whose friends are applying to go abroad with greater ambitions, practicing in rural areas of Nepal is not an attractive choice.” Money was highly important, but other issues also rated – “There is no point in having high salary in rural places if you don’t have things to spend that money on.”

## Conclusions

In a country in need of generalists, this study has confirmed the general low interest and knowledge about general and rural practice among medical students and young doctors in Nepal. One of the main challenges is to raise the profile of and awareness of generalists for Nepal within the medical community, in the government and among the general population. There appears to be opportunity to influence career choice during undergraduate training. In line with WHO recommendations [2,3], curricula changes are needed to include rural health topics, to provide greater exposure to general rural practice (both rural community experiences and clinical rotations) with good role models, and to enhance the competencies of health professionals working in rural areas.

There is clearly a need to increase the exposure to and the recognition of general practice through provision of incentives and attractions for general practice postgraduate training and later service. In line with WHO recommendations [3], these should include:

• scholarships, bursaries or other education subsidies with enforceable agreements of return of service in rural or remote areas;

• combinations of fiscally sustainable financial incentives, personal and professional support including improved living conditions, a good and safe working environment, skilled mixed teams and cooperation between health workers from better served areas and those in underserved areas;

• career development programmes and public recognition measures such as rural health days, awards and titles at local, national and international levels to lift the profile of those working in rural areas.

For diplomas to be a useful added academic qualification they must have recognition. Targeting the MBBS 2 year government bondholders for distance courses may be a way of pioneering this in Nepal.

### Limitations

Sixty-two percent of students and young doctors returned a questionnaire. It is likely that the respondents were biased towards those more interested in both completing the forms and taking part in focus groups. This may have been particularly so in the resident/intern group from whom there was a much lower response (about 40%) and particularly for those from out-of-Kathmandu colleges (about 28%). The focus groups only included a small number of medical students and doctors from six of Nepal’s medical schools and may not represent all medical schools.

This study looked only at intentions rather than demonstrated actions. It is possible there may be an element of giving expected answers rather than expressing actual plans. However, the questionnaire was anonymous.

Also, since this was a cross-sectional study, it only gives a snapshot of choices and influences at one point in time. A longitudinal study that follows the same students from the beginning of medical school through their training and to their ultimate career choice would give more robust information about what affected their career choices.

## Abbreviations

BPKIHS: BP Koirala Institute of Health Sciences; CME: Continuing Medical Education; GP: General practitioner; IOM: Institute of Medicine; MDGP: General Practice Postgraduate Programme (Nepal); MBBS: Bachelor of Medicine and Bachelor of Surgery; MD: Masters in Medicine; MS: Masters in Surgery; MPH: Masters in Public Health; NAMS: National Academy of Health Sciences; PG: Postgraduate; SLC: School Leaving Certificate; WHO: World Health Organization

## Competing interests

Both authors work with the Nick Simons Institute (NSI) which fully funded this study and is involved in training and supporting rural health workers in Nepal. BH is a volunteer consultant and RS is an employee of NSI. Both authors are involved in NSI’s scholarship programme for MDGP (postgraduate General Practice). BH is the Professor and Coordinator of the National Academy of Medical Sciences (NAMS) MDGP programme in Patan Hospital.

## Authors' contributions

BH conceived of the study, participated in its design and coordination, collected data, performed statistical analysis, interpreted data and drafted the manuscript. RS participated in its design and coordination and collected and interpreted data. Both authors read and approved the final manuscript.
